# 

**Published:** 1841-08

**Authors:** 


### CONTENTS

OF NO. II.

### ORIGINAL COMMUNICATIONS.

### ESSAYS AND CASES.

ART. I.—An Address on the Improvement of the Medical Profession; delivered before the Medical Society of Tennessee, at its twelfth annual meeting, in Nashville, in May, 1841. By LUNSFORD P. YANSDELL, M. D., Prof. of Chemistry and Pharmacy in the Louisville Medical Institute. - - - - - - 81

ART. II.—Case of Disunited Fracture successfully treated with the Seton. By WM. H. DONNE, M. D., of Louisville, Ky. Reported by A. Martin, resident student in the Louisville Marine Hospital. - - - 97

ART. III.—Objections to the Operation for Hydrocele by Injection, in comparison with that by Incision. By THOMAS CARROLL, M. D., of Cincinnati, Ohio. - 100

ART. IV.—Cases of Fracture and Dislocation, reported to the Medical Society of Tennessee, in May, 1841. By FELIX ROBERTSON, M. D., of Nashville, Tenn. 106

ART. V.—A Case of Hydrocele, reported to the Medical Society of Tennessee at its annual meeting in May. By FERDINAND SMITH, M. D., of Franklin, Tenn. - 107

Art. VI.—Operation for Imperforate Vagina. By John Travis, M. D., of Carroll county, Tenn. - - 110

REVIEWS.

Art. I.—Traite des Maladies Ventenses, ou Lettres sur les Causes et les Effets de la Presence des Gaz ou vents dans les voies Gastriques, et sur les Moyens de Guerin ou de Soulager ces Maladies; par M. P. Baumes, Chirurgien en chef de l'Hospice de L'Antiquaille de Lyon, &c. &c. 111

SELECTIONS FROM AMERICAN AND FOREIGN JOURNALS.

Operations for the Cure of Stammering. - - 125
Observations on Vaccination and Small Pox, &c. 135
Results of Re-vaccination in the Deaf and Dumb Institution of Paris. - - - - - - 138
General Result of the Vaccinations and Re-vaccinations in France. - - - - - - 139
Rupture of the Fallopian Tube from accumulation of the Catamenial Fluid. - - - - - - 141
Obliteration of the Inferior Vena Cava. - - 142
Wound of the Heart. - - - - - 144
Case of Punctured Wound of the Ascending Aorta, fatal in fifteen minutes. - - - - - - 146
Ligature of the common Carotid Artery, and Experiments on the Ligature of both Carotids in Animals. - - - 148

ORIGINAL INTELLIGENCE.

The Leviathan. - - - - - 149
Medical College of the State of South Carolina. - - 156
Death of Professor Wagner. - - - - 157
Medical College of Tennessee. . - - - 157
The Vapors of Nitrate of Potassa in Asthma. - - 158
Tubercles Developed by Intermittent Fever. 159
Lithotomy. - - - - - - 159
The Weather. - - - - - - 160
The Louisville Medical Institute. - - - - 160
Dunglison's American Medical Library. - - 160

